# Natural Fibre-Reinforced Polymer Composites (NFRP) Fabricated from Lignocellulosic Fibres for Future Sustainable Architectural Applications, Case Studies: Segmented-Shell Construction, Acoustic Panels, and Furniture

**DOI:** 10.3390/s19030738

**Published:** 2019-02-12

**Authors:** Hanaa Dahy

**Affiliations:** 1 BioMat Department: Bio-Based Materials and Materials Cycles in Architecture, at ITKE: Institute of Building Structures and Structural Design, University of Stuttgart, Keplerstr. 11, 70174 Stuttgart, Germany; h.dahy@itke.uni-stuttgart.de; Tel.: +49-(0)711-685-832-74; 2 Faculty of Engineering, Department of Architecture (FEDA), Ain Shams University, 11517 Cairo, Egypt

**Keywords:** biocomposites, NFRP, segmented shell, multi functionality, acoustic absorption, furniture, design for sustainability, post-fabrication, lignocellulosic-based composites, sustainable architecture, extrusion, resin-bath, molding

## Abstract

Due to the high amounts of waste generated from the building industry field, it has become essential to search for renewable building materials to be applied in wider and more innovative methods in architecture. One of the materials with the highest potential in this area is natural fibre-reinforced polymers (NFRP), which are also called *biocomposites*, and are filled or reinforced with annually renewable lignocellulosic fibres. This would permit variable closed material cycles’ scenarios and should decrease the amounts of waste generated in the building industry. Throughout this paper, this discussion will be illustrated through a number of developments and 1:1 mockups fabricated from newly developed lignocellulosic-based biocomposites from both bio-based and non-bio-based thermoplastic and thermoset polymers. Recyclability, closed materials cycles, and design variations with diverse digital fabrication technologies will be discussed in each case. The mock-ups’ concepts, materials’ compositions, and fabrication methods are illustrated. In the first case study, a structural segmented shell construction is developed and constructed. In the second case study, acoustic panels were developed. The final case studies are two types of furniture, where each is developed from a different lignocellulosic-based biocomposite. All of the presented case studies show diverse architectural design possibilities, structural abilities, and physical building characteristics.

## 1. Introduction

The building industry accounts for more than 35% of global final energy use, nearly 40% of energy-related CO_2_ emissions [1], and almost 45% of global resources’ consumption. As the global population will grow from seven billion to almost nine billion by 2040, the demand for resources will rise exponentially, and by 2030, the world will need at least 50% more food, 45% more energy, and 30% more water; at the same time, environmental boundaries will have constrains on supply, especially with climate change [2].

The continuous global population growth means the need for new housing units in a synchronised velocity to the population growth. In Germany alone, there is an estimated need for between 350,000–400,000 new dwellings to be built every year [3]. To meet this demand, further non-renewable resources will be consumed, leading to severe environmental destruction if the same types of building materials resources are applied.

Aggregate materials and concrete are the predominant building materials by weight used in Europe at the moment, which together with high-emission materials such as steel and aluminum are responsible for the largest share of greenhouse gas (GHG) emissions stemming from the building sector [4]. To replace these classic materials, several developments took place in the building materials of the future, including lightweight materials branch called fibre-reinforced polymers (FRP). These are often referred to as (composites), which are composed mainly of fibres bonded by a binding material. These FRP materials can induce lightness, hence reducing the need for concrete and metals in construction, and allowing another level of geometrical variations and integrated functionalities in building systems. However, these materials still depend mainly on non-renewable fossil-based materials such as carbon and glass-fibres, which would still not solve the original problem of resources and CO_2_ emissions. Hence, replacing this solution through natural fibres (NF) instead of synthetic fibres to develop natural fibre-reinforced polymers (NFRP), which are also named (biocomposites), could offer a solution to the building sector and be a vital suitable building material of the future. Lignocellulosic natural fibres based on abundant annually renewable resources such as recycled agricultural residues (agrofibres) e.g. straw, as well as other industrial lignocellulosic natural fibres such as flax, jute, and hemp can be applied as main components in the production of biocomposites. Biocomposites can also be used to replace wood applications in the building industry, hence improving forestry practices and reserving such a vital slow renewable resource that plays a main ecologic role on our planet. In addition, several innovative building systems can be developed completely based on biocomposites applications using various production/fabrication techniques.

Accordingly, diverse case studies have been developed in both structural and non-structural architectural applications, which are hereby illustrated and analysed in this paper, offering multi-functional solutions of biocomposites in the building industry. In the highlighted developments, the matrices play an important role to offer recyclability options, when the organic binder applied is in the form of thermoplastic polymers; whereas smart designs are applied to guarantee reusability when the binder applied is a thermoset polymer. In all the cases, fabrication technologies dependent on the matrix type applied and the physical form of the lignocellulosic fibre played an important role to guarantee the closed-material cycle conception, whether through recyclability or reusability. The highest efficiency of lignocellulosic fibres to be applied in the building industry depend on the possibilities of using those resources as a main component in biocomposites in the form of fibres, polymers, or both. In the following, further emphasis on this last-mentioned point will take place.

## 2. Extraction of Lignocellulosic Fibres and Biopolymers from Annual Lignocellulosic Resources

Depending on the main components of the lignocellulosic fibres, fibrils could be extracted and applied as a main biocomposite ingredient. In most of the lignocellulosic fibres collected from stalks to produce long endless fibres such as hemp, jute, ramie, and kenaf, the fibre is integrated with natural gum in the plant structure. Removal of this gum is made in a process called “retting” [5], where bacteria and natural-occurring fungi is applied to remove lignin, pectin, and other impurities on the cellulose fibres that need to be extracted. This conventional method takes a lot of time; therefore, for economic reasons and not environmental ones, chemical methods mostly replace natural methods, especially using sodium hydroxide (NaOH), or through mechanical methods using decorticating machines, steam explosion (STEX), and ammonia–fibre extraction methods as well [6].

Biopolymers could be extracted as well, where the glucose derived from cellulose could be transformed to lactic acid, and accordingly to PLA (poly lactic acid) synthesis. Similarly, lignin can be extracted to formulate lignin binder, which can be plasticised to act as a thermoplastic against its original feature as a thermoset [7]. Bioresins could be synthesised from plant oils and hemicellulose derivatives. Accordingly, different bioplastics as well as bioresins, in addition to lignocellulosic fibrils, which are the main biocomposite components, can all be derived from the same source to be modified and compounded offering different application possibilities, as shown in this paper. Fibres and polymers extraction possibilities from lignocelluloses are given in Table 1.

Economically, the bioplastics prices gap decreased in comparison with fossil-based plastics. PLA is one of the dominating bioplastics in the contemporary markets as well as Bio-HDPE (bio-based high-density polyethylene), where both compete price-wise with conventional petro-based polyolefins.

All of the previously mentioned extracted materials can be set as components in biocomposites or green biocomposites, in case both the fibres and the matrix were biomass-based.

## 3. Lignocellulosic Biocomposites

### 3.1. Lignocellulosic Thermoplastic Biocomposites and WPCs

One of the successful and wide applications of natural fibres with plastics is the wood plastic composite (WPC), which started in Europe as a leader in this industry since the end of 1990s [10]. WPCs appeared historically much earlier in the form of milled wood-filled thermosets before the 1960s, and started appearing in its known wood plastic composite form only in the 1990s [11]. The chopped or milled wood fibre can be mixed up until 90% by weight to plastics [12], which are normally PP or PE (polyolefins) or PVCs (PolyVinyl Chloride); however, the typical wood plastic product is a mixture of 70% wt. of wood flour with 5% additives for compatibility as bonding agents, in addition to UV-protection additives as well as pigments, which are all added to the 25% plastic [10].

In many commercial WPC products, lignocellulosic fillers range from 35% to 60% only, depending on the mark type and the required properties. WPC is not limited to wood lignocellulosic filler’s appliance, but rather a wide range of natural fibres’ appliance possibilities, including lignocellulosic fibres, especially agricultural by-products [11].

The combination of the high lignin-filled wood dust together with other flammable plastics, such as polyolefins, increase the critical situation of WPCs’ future appliances in buildings, due to the new fire-resistance regulations set worldwide. Solving this through classic mineral additives should be theoretically possible, as Klyosov described in his book, but in this case, the final price will highly increase, which is already around two to three times the normal wood price, and the WPC will turn into a mineral-filled plastic instead of a wood-fibre filled one. Most of the WPCs lie in the material category ‘C’ of the FSI (flame spread index) lying between 76–200, in comparison to normal wood, which has an FSI ranging from 100 to 200. The combination of WPCs with low-flammable plastics such as PVCs results in WPCs with a lower FSI range of 25–60 [10]. However, the appliance of PVCs is still environmentally criticised. Therefore, replacing WPC with lignocellulosic fibres with high mineral contents and lignin can allow a wide range of applications in architecture with improved fire class and flammability behavior [7].

Many investigations took place with respect to non-wood lignocellulosic fibres compounded with fossil-based plastics whether in Europe, North America, or Asia, resulting in products that resemble WPCs (wood plastic composites) in physical and mechanical properties, providing the high optical, free-design possibilities, and the ability to be colored and molded in different geometries according to the desired consumer requirements. This helped in replacing WPCs through the newly evolved biocomposites in the contemporary building markets.

Replacing wood and other industrial natural fibres became a big interest in many fibre-based industries mainly for economic reasons, due to the low cost of the worldwide available agricultural by-products such as straw, which ranges between 40–100 Euro/ton depending on the location, quality, and densification applied (whether baled, chopped, or pelletised) [8].

The application of lignin—the natural organic cement-like polymer of plant cellulosic fibres that is responsible for the plants’ stiffness—as an isolated applied thermoplastic biopolymer of its own as declared in Table 1, has been rarely recorded.

In previous research work by the author [7,13], straw was filled in a modified thermoplastic lignin instead of the cellulose fibres, and the properties’ differences between the two green lignin biocomposites were analysed, especially for the fire-resistance behavior. The result has shown a large improvement due to the application of raw rice straw that is naturally filled with silica, causing a change in the material class of the straw–lignin biocomposite. This proposes those types of materials strongly in interior finishings.

### 3.2. Lignocellulosic Thermoset Biocomposites

Straw thermoset biocomposites form another important application possibility of straw in other innovative applications in architecture, offering new design options as well as other important economic advantages, replacing commodity fossil-based products, which are usually applied externally.

The validity of natural fibre thermoset composites had already been proven, especially in the direction of automotive applications, whether for building up interior parts of the vehicles or even exterior complete vehicles’ bodies [14]. For example, hemp and soya-oil derivatives have been applied to produce high-quality and low-weight car bodies. Similarly, different types of natural fibres have been introduced in the same industry to reduce both the weight and cost of the final product. Famous industrial natural fibres, including hemp and flax, overwhelm such industries, as previously described. However, recently, the importance of giving straw and agricultural by-products the chance to prove their potential in the same industry has been highlighted through combining straw with thermoset polymers to manufacture value-added, ultra-light composite products for the automotive and transportation industries. The high value of the special inner chemical structure of the straw on the improved behavior of the final product is based on the vascular bundles and central void, the presence of microfibrils in the structure, and the existence of lignin polymer near the surface of the straw. The team in this study suggested after previous work on lignin’s effect on the interface bonding value of the straw fibre/matrix that under appropriate conditions, lignin’s chemicals diffuse to the epidermis surface, thus forming cross-links with thermoset polymers. This theme illustrates the high potential of straw in biocomposites, when its inner components would be properly studied and well used in biocomposites’ scope.

## 4. Fabrication Possibilities

### 4.1. First Fabrication Phase of Semi-Finished Products: with Thermoplastic and Thermoset Binders

Biocomposites’ processing technique depends mainly on the type of binder/matrix applied and the physical form of the natural fibre. In the case of lignocellulosic agro-based bast fibres, fleece and mats’ making are options for forming textile-wise forms of the agrofibres that can optimise their application varieties. However, at the moment, the physical forms of these types of agrofibres do not exist in industrial scales for industrial applications of biocomposites. The commercial lignocellulosic agrofibres biocomposites depend mainly on short fibre processing techniques, including compounding, extrusion, and molding. Foaming techniques for short natural fibres’ polymeric composites were also found to be possible at the laboratory scale [15].

The common compounding process takes place through double screw extruder machines by heating the thermoplastic polymer, whether externally or through the mechanical shearing of the inner screw(s). Such processing is limited to the short fibres’ compounding. Long and continuous fibres are mostly not applied. However, in recent research studies, this became of high interest and started at lab scales. The heating temperature should be carefully applied, and should not exceed a certain limit (around 190–200 degrees), so as not to cause the rapid decomposition of the fibre. This would lead to failure in the compounding process and /or unsatisfactory composite performance.

**Figure 1:**
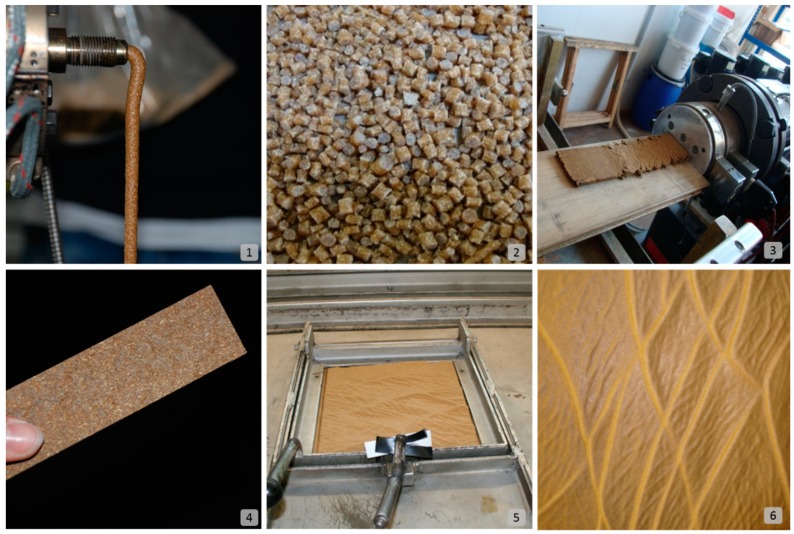
Up: (**1**) Compounding, (**2**) pelletising, and (**3**) extrusion processes. Down: (**4**) Cut panel, (**5**) the thermoforming-process of a thermoplastic biocomposite panel, and (**6**) a thermoformed final finished panel (photos: Dahy, H.; republished [8]).

Generally, thermoplastic biocomposites can be either directly extruded into a final product through a mould that should be fit in the machine in this case; alternatively, they can be pelletised and packed for a reprocessing procedure through further extrusion, as shown in the upper images of Figure 1 (from stages one to three), or injection in a separate process. In other types of lignocellulosic natural fibres gained from fibre crops, such as flax, hemp, kenaf, and cotton, other compounding methods can be applicable, as the fibres in this case are long enough to produce yarns and fabrics; then, comingling processes are possible. The composites in this case are finalised through hot pressing so that the polymer would diffuse within the fibres, resulting in a high-performance natural fibre composite in case of applying thermoplastics.

Biocomposite thermosets are usually processed through the closed molding technique, applying pressure and vacuum. In this case, the production of semi-finished thermoset products in this case does not usually need complex or heavy machinery, unlike the processing methods using thermoplastic polymers. However, the main obstacle is the non-recyclability, due to the presence of the thermoset resin in the biocomposite mixture. Downcycling through chopping and incineration to recover applied energy are applicable. In addition, reusability options using smart designs and smart connection systems should be applied to allow guaranteed closed-material cycles.

### 4.2. Second Processing Phase: with Thermoplastic and Thermoset Binders

After processing, thermoplastic biocomposite products are finished through lamination, thermoforming, drilling, and other processes to give the product its final design and attractive touches, as shown in the lower images of Figure 1 (from stages four to six). This aspect is important in all industrial applications, especially in architectural ones. On the other hand, lamination, molding, pressing, Computer Numerical Control (CNC)-drilling, and other post-processing methods take place in the case of applying thermoset binders.

Throughout the following case studies, diverse post-processing techniques are applied on the semi-finished lignocellulosic-based biocomposites to enhance several multi-functionalities, whether structural, acoustical, or others.

## 5. Applications

### 5.1. Case Study One: Segmented Shell Construction with Thermoplastic and Thermoset Binders (Biomat Research Pavilion 2018)

**Figure 2:**
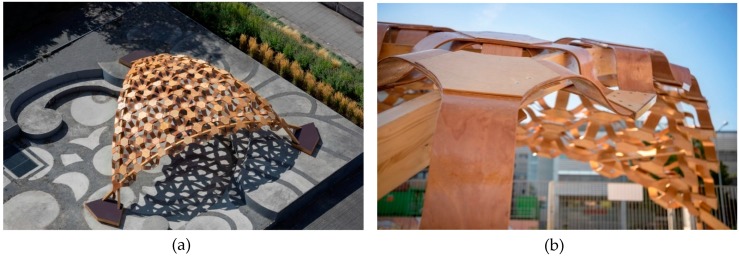
(**a**) The erected BioMat pavilion at the campus of University of Stuttgart; (**b**) Connections of modular biocomposite sandwich panels in combination with the structural beams. © BioMat/ ITKE-University of Stuttgart.

The BioMat research pavilion 2017/2018 is a 3.6-metre-high shell structure with a 9.5-m span, covering an area of 55 m². It was located on the campus of the University of Stuttgart in the city centre between August–December 2018, as shown in Figure 2. Its special feature is the modular construction of lightweight, single-curved elements that form a double-curved shell with individual segments consisting of veneer-reinforced elastic biocomposite cores. The structure is supported by three crossed beams.

The elastic biocomposite boards that were applied in the core of the biocomposite laminated panels were exceptionally elastic to enable the fabrication of extremely double-curved surfaces for the construction of the final shell. The post-fabrication techniques took place where the elastic biocomposite core was numerically CNC-milled according to the set parametric computational model, and was stiffened through lamination with veneer in a vacuum press bag, with no heat or humidity pre-treatment. The resulted engineered veneer-reinforced biocomposite was adapted to higher stresses, and was applied as a structural component in the segmented shell construction. The pavilion has allowed the testing of the whole developed building system under real conditions.

**Figure 3:**
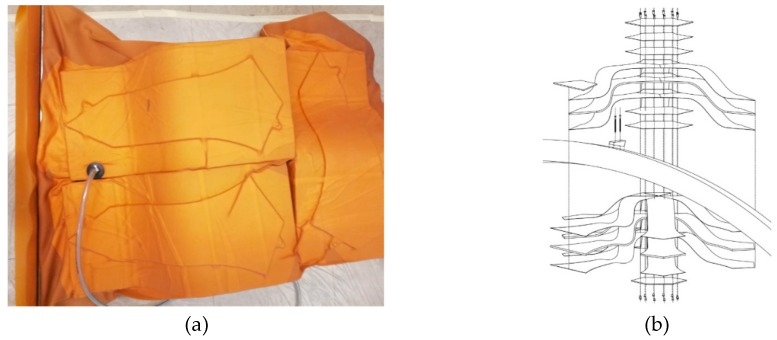
(**a**) Vacuum-pressing fabrication technique applied in the production of the sandwich panels using the closed molding technique; (**b**) Isometric view of single layers and connections of one of the four details of the applied modular system. © BioMat/ITKE-University of Stuttgart.

The key advantage of using this material is the easy possibility of adjusting the mechanical properties along with the diverse needed geometrical variations. Through the arrangement of the special veneer reinforcing materials—known as 3D veneer—with multiple thermoplastic reinforcing perpendicular ribs on the backside of the longitudinal arranged veneer strips, the structural behaviour of the veneer laminated lignocellulosic biocomposite and wooden sandwich panels were improved. Accordingly, the needed target mechanical properties were reached. The modular elements of the pavilion were single-curved fabricated, and the arrangement of the elements’ layers and connections through the smart settled design and the chosen smart numbering system, together with the data management that was applied, enabled the final double-curved shell to be successfully constructed, meeting the highest possible accuracy within the construction phase. Diverse detailing solutions for the layering of the biocomposite sandwich panels and their connection systems have taken place. In Figure 3, one of the four main detail solutions of the pavilion is illustrated.

The parametric form-finding process enabled the possibility of finding the proposed architectural solution and fabrication system, where 121 prefabricated components were assembled in four large triangles on site, and then lifted into the correct position in space to form the final large-sized double-curved triangular form. The smart design and the applied connection system guaranteed the possibility of reusing the single segment units to suit further construction designs and other constellations. This was set in the initial design phase to guarantee a closed-material cycle of the constructed temporary building.

### 5.2. Case Study Two: Acoustic Panels with Thermoplastic Binders

In the framework of an industrial project named “PLUS: Environmentally-friendly sandwich panels”, it was planned to produce sandwich panels with integrated thermal insulation and acoustic absorption functions. However, it was only possible within this project to produce one type of foamed bioplastic core with closed celled pores through the research group of foams in the Fraunhofer Institute for Chemical Technology, which was one of the project partners. This foam type was responsible for the thermal insulation, and successfully, a thermal conductivity of ca 0.035 W/m*K of the newly developed biofoams was reached [16]. To reach the acoustic absorption that was needed, lignocellulosic fibre-based composites, based on the patent [17] by the author and upgraded to have semi-elastic and hard fibreboards after combining PLA as a main component ranging from 10% to 60% by weight, were applied in the skin outer layer of the developed environmentally-friendly sandwich panels. The new recipes created a new class of materials that was capable of being thermoformed to achieve different air cavities trapped behind the first skin surface of the sandwich panel. Those air cavities created a phenomenon known in the acoustics field as resonances. Therefore, cavity resonances were created artificially through the post-processing of the outer sandwich panels skins, and these absorber systems were set in combination with the classic porous absorbers systems. In cavities, depending on the geometry at different frequencies, resonances occur, which are due to the intrinsic oscillations of the hollow space. The sound absorption of multiple shelled sandwich elements depends largely on its position and distance from the back wall on which the sandwich panels are mounted. The test occurred once with the elements placed directly on the wall and once with the elements at a distance of 50 mm from the back wall. The test was done using an impedance tube following the standard ISO 10534-2:1998 (Acoustics – Determination of sound absorption coefficient and impedance in impedance tubes – Part 2: Transfer function). The results of the measured sound absorption degrees depended on the frequencies, ranging from 100 to 2500 Hz, as well as the corresponding rated sound absorption grades, ranging from zero to one.

#### 5.2.1. Ignot Acoustic Panel

**Figure 4:**
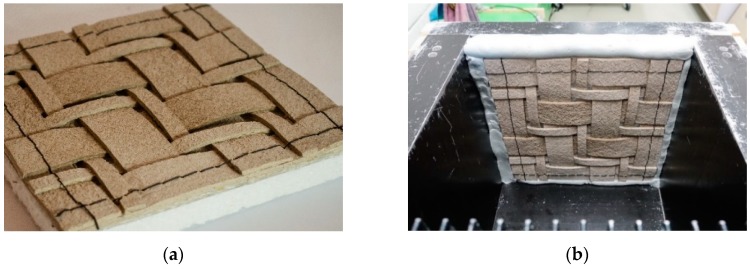

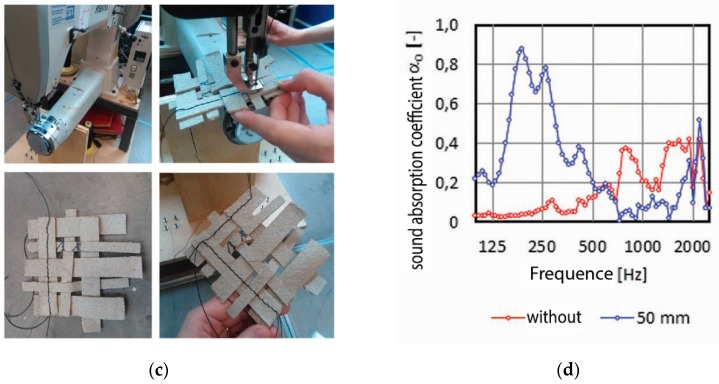
Ignot bio- acoustic sandwich panel (**a**) bio-acoustic sandwich panel before acoustic measurement (**b**) bio-acoustic sandwich panel during the acoustic absorption test with closed edges; (**c**): Knitting/tailor process of the lignocellulosic biocomposite layers before being fixed on the biofoam core, Photo: Subaciute and Stankaityte, BioMat-ITKE/University of Stuttgart; (**d**) Compilation of the measured sound absorption grades dependent on the frequency, as well as the evaluated sound absorption grades of the Ignot sandwich elements from variants zero to one.

The panel was manufactured through cutting the lignocellulosic composite panels developed in the PLUS project and tailored in a geometrical order with an industrial six-axis robotic head, guaranteeing the textiles in the outside surface being set as connectors. The acoustic absorption is improved upon fixing the panel 50 mm away from the back wall, as shown in Figure 4d. In addition to the acoustic absorption, thermal insulation was also reachable in the same panel. Furthermore, improvement in the acoustic absorption of the developed panel could be guaranteed through adding a damping material of thin lignocellulosic textile layer between the tailored biocomposite layers and the biofoam core.

#### 5.2.2. Polycal Acoustic Panel

This biocomposite acoustic absorber was very successful in the evolved results, as it was a mixture between a cavity resonator and a porous absorber. The two outside layers of the semi-finished thermoplastic NFRP panels were thermoformed, producing the needed cavity of encapsulated air that is necessary to absorb the waves through damping and diffusing the acoustic energy into kinetic energy creating resonance. The presence of the absorbing layer of the fleece made from hemp 65%, kenaf (25%), and Polyethersulfone (PES) (10%) from J.Ditrich and Söhne Vliesstoffwerk GmbH in the core of the panel created a big improvement in the absorption class that was reached.

**Figure 5:**
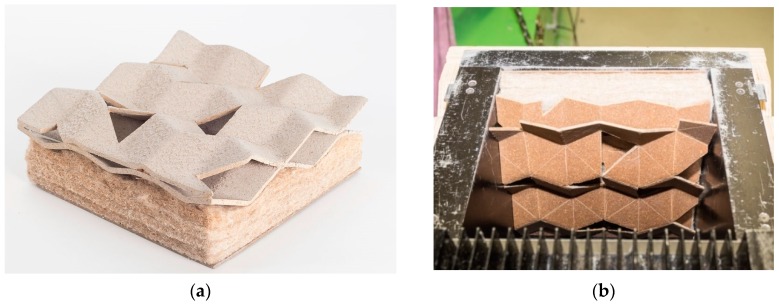

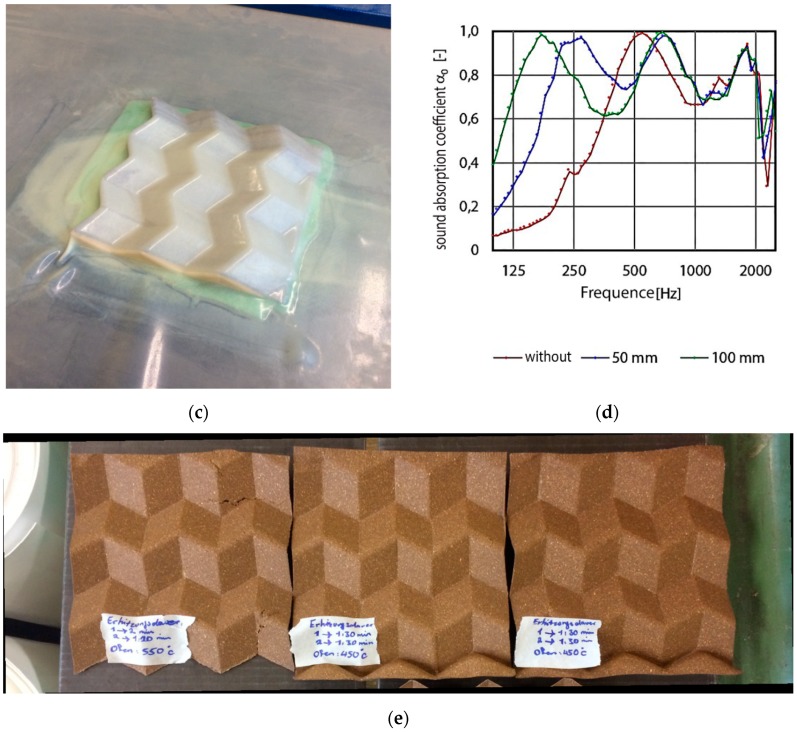
(**a**–**d**): Illustration of the fabrication methods and the acoustic absorption graph of the *Polycal* acoustic panel; (**e**): Diverse tests that took place to specify the processing temperature of thermoforming techniques. Photo credit: Banaditsch, Foroutan & Moradian, BioMat-ITKE/University of Stuttgart.

In Figure 5, the fabrication methods of the outside layers of the sandwich panels experimenting with thermoforming at different temperatures are illustrated. In the acoustic absorption graph, two resonance tips were already reached without wall spacing. By varying the wall distance, a third resonance maximum was deduced; thus, the best effect to absorb a broadband was reached.

### 5.3. Case Study Three: Furniture from Biocomposites 

In this section, two types of biocomposites are illustrated through which two variables of furniture were fabricated. The first chair/lounge is from thermoplastic-based biocomposites using short lignocellulosic fibres, while the second chair is from thermoset-based biocomposites using endless lignocellulosic fibres. A detailed analysis is illustrated as follows.

#### 5.3.1. Plus Lounge Furniture from Biocomposites with Thermoplastic and Thermoplastic Elastic Binders (Tpe)

**Figure 6:**
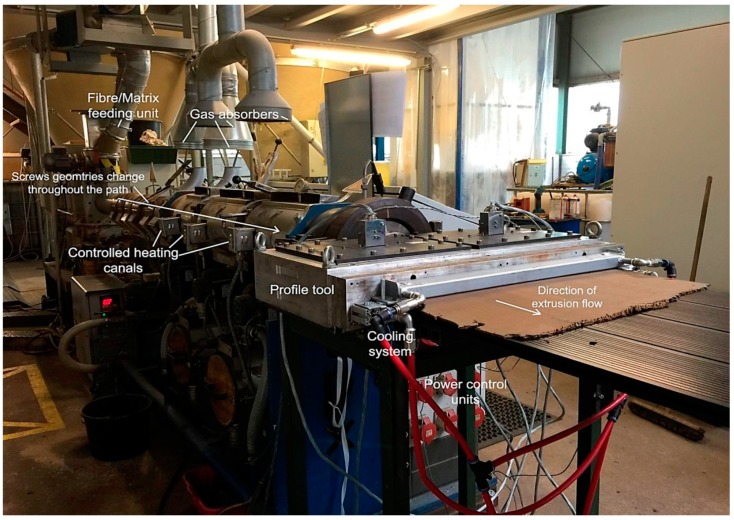
Illustration of the production of Bio-flexi in the mass-production scale indicating the control and feeding units in Naftex GmbH company, Wiesmoor, Germany (Photo: Dahy, H. republished [7]).

**Figure 7:**
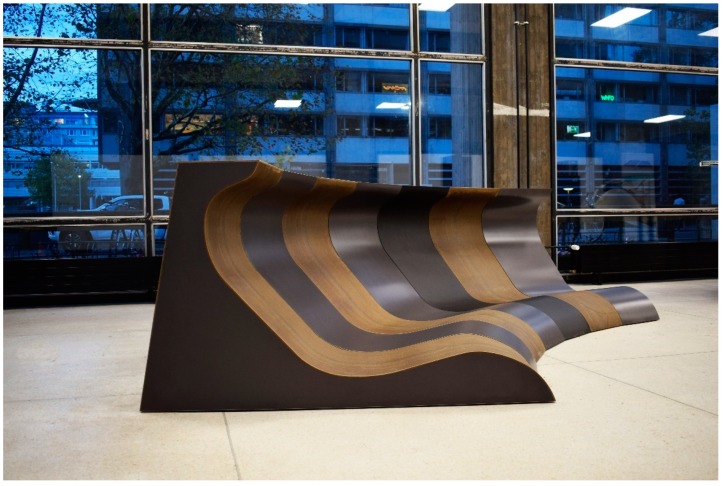
The lounge exhibited in the foyer of the faculty of architecture in the University of Stuttgart, Germany. Photo © BioMat at ITKE, University of Stuttgart.

The lignocellulosic composite recipes used here were based on the patent [17] of the author and further developed into three successive research projects, namely: “PLUS”, “Bioprofile”, and “Bio-Faserplate”, which was led by the author too. The recipes varied between mixtures of straw and other lignocellulosic fibres in combination with bio-based HDPE, classic petro-based HDPE, and PLA separately. In combination with a thermoplastic elastic binder to adjust the elasticity, the geometrical flexibility of the extruded panel was reached. The panels were extruded using a twin-screw natural fibre extruder machine after adjusting the controlling conditions. Among these, gas absorbers were placed for excess vapors from the natural fibre existed, as shown in Figure 6, to guarantee the quality and avoid having air bubbles in the cross-section of the panel after extrusion. The panels were supported on thin wooden frames and did not need further modification of heat adjustments for thermoforming as in the previous example (5.2). The design of the shown lounge in Figure 7 was developed by the author and her research team. The outcome was exhibited in the foyer of the Faculty of Architecture in Stuttgart in 2017, in the Hannover Exhibition, and in the Composites Europe Exhibition in Stuttgart in Germany, which were both in 2018.

#### 5.3.2. Hemp Chair: Furniture from Biocomposites with Thermoset Binders

**Figure 8:**
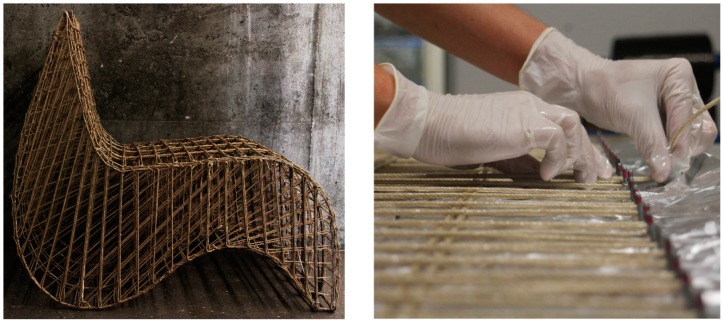
The hemp chair during production, with an emphasis on the winding process applied for the lignocellulosic endless fibres that are pre-soaked in the resin bath. Photo credit: Sachin and Kauffmann, BioMat-ITKE/University of Stuttgart.

This example shows another variation of combining lignocellulosic fibres with biopolymers. The difference here is in the physical form and type of the applied natural fibres (endless hemp fibres present in the form of yarns) passing in epoxy resin bath, and then stretched between nails fixed in a wooden board, having the silhouette of the chair cross-section, in a process known as “coreless filament winding”, as shown in Figure 8. The fibres were first tested mechanically; then, the resulted E-Modulus (*ca*. 5 GPa) was used to adjust the best form of the chair reaching the optimum equilibrium state. Structural simulations and buckling mode analysis took place through finite element methods for beam elements using the Grasshopper plugin Karamba and the software Sofistik.

**Figure 9:**
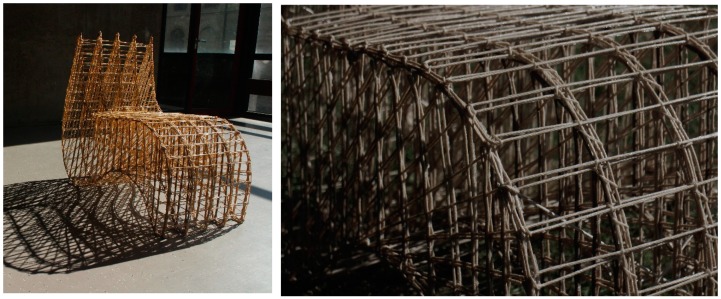
The final hemp chair with a closer illustration of the detailing of the connection. Photo credit: Sachin and Kauffmann, BioMat-ITKE/University of Stuttgart.

The lignocellulosic hemp fibres were left stretched, and tension forces were applied through adjustments at the anchor points until the fibre–matrix composite completely solidified. Extra zip-ties were bound on the fibres surfaces, and pressure was applied to prevent the fibres from movement during the crystallisation process and to avoid delamination. In a separate process, two flat sheets were designed and fabricated from the same materials; then, they were bent to act as a structural balancing closure to the chair profiles from up and down, forming the seating surface from above and the bottom of the chair from below, as shown in Figure 9. The separate chair sections were brought into an equilibrium state through combining the upper and lower actively bent sheets with the longitudinal chair profiles. This was done manually through weaving fibres that also passed in a resin bath combining the longitudinal profiles and the sheets. Two X-bracings were adjusted underneath the seating area, at the back and in the front edge underneath the seating area to guarantee lateral resistance and avoid distortion.

## 6. Conclusion and Discussion

Through the illustrated case studies of NFRP/biocomposites, a thorough and deep proof was given of the possibility of a broad appliance of these newly developed sustainable materials with various fabrication techniques and diverse designs in multiple architectural applications. Thermoplastic biocomposites have the privilege of offering diverse closed material cycle possibilities, including recyclability and reusability. The fabrication and processing techniques of thermoplastic biocomposites require high temperatures and energy consumption. High initial investments for classic compounding machines are usually needed in this case, but the large mass production possibilities and guaranteeing recyclability are major advantages from this side. Digital fabrication technologies such as 3D printing and additive manufacturing techniques can overcome the large investment costs of the classic thermoplastic compounding and extrusion techniques.

On the other hand, thermoset biocomposites propose a variety of design possibilities, large-scale applications, and higher mechanical properties. Larger labor and lower machinery investments are needed in this case. However, digital fabrication technologies for processing and post-processing techniques and automated manufacturing methods can wisely overcome the large labor costs that are needed.

Aside from the fabrication techniques and their advantages and disadvantages, this paper has shown the wide application possibilities of this branch of sustainable building materials, NFRP, which act as a direct alternative to diverse non-renewable resource-based building materials.

To reach optimised solutions in future sustainable building practices, biocomposite materials need to be synthesised using digital fabrication technologies and computational design applications to enable a rapid replacement of fossil-based building elements and cope with the high need of increasing the production speed for future building elements. Through this, a holistic strategy for rethinking sustainability in future architecture needs to be created in order to fit in the rapid coming change in the built environment.

This applied strategy of alternative sustainable building materials’ applications serves the crucial need of decreasing the environmental destruction and global warming that partly resulted from the cradle-to-gate production of the current materials used for buildings [4]. This will not only meet the velocity challenge of the future construction processes, it will also guarantee that the resources applied are based on the optimum social, ecologic, and economic values that need to be met. Linking this approach to the set European Union (EU) Bioeconomy Strategy [18], with the main focus on bio-based resources as well as on sustainability and cascading the use of biomass, matches the high need of solving the current problems of resources and energy as well as improves the current practices in the building sector. The proposed bio-based solutions in this article enhance complete closed material cycle and recyclability/reusability options. This will decrease the overall construction and demolition wastes (C&D) matching the EU bioeconomy and circular economy strategies for the future.

## Figures and Tables

**Table 1:** Illustration of the fibres and polymers’ extraction possibilities from the lignocellulosic fibre component ([8,9]). PLA: poly lactic acid.

Lignocelluloses	
Fibre Extraction	Biopolymer Synthesis
Fibrils extraction (mechanically, chemically, through bacteria, others)	Cellulose > Glucose > Lactic acid/PLA
Fibres direct usage (bales as building units)	Hemicellulose > Xylose > Xylit/Furan resin
Fibres densification and geometrical modifications (fibre mats, yarns, pellets, chopping, hybrids, others)	Lignin > Lignin/Phenols > Polymers
